# *Cirsium arvense* (L.) Scop.: Phytochemistry, Traditional Uses, Pharmacological Activities, and Future Therapeutic Potential

**DOI:** 10.3390/plants15121835

**Published:** 2026-06-13

**Authors:** Kairat S. Zhakipbekov, Murat Z. Ashirov, Galiya Z. Umurzakhova, Elmira N. Kapsalyamova, Azhar Y. Omirbayeva, Farida E. Kayupova, Klara Z. Zhumalina, Aigul G. Ibragimova, Elmira A. Serikbayeva, Ardak B. Bakytzhanova, Amina D. Farkhatova

**Affiliations:** 1 School of Pharmacy, Asfendiyarov Kazakh National Medical University, 94, Tole Bi Str., Almaty 050012, Kazakhstan; zhakipbekov.k@kaznmu.kz (K.S.Z.); ashirov.m@kaznmu.kz (M.Z.A.); elmira_kaps@mail.ru (E.N.K.); farhatovaamina68@gmail.com (A.D.F.); 2 Faculty of Pharmacy, South Kazakhstan Medical Academy, Al-Farabi Square, 1, Shymkent 160019, Kazakhstan; galiaum@mail.ru (G.Z.U.); simonmed@mail.ru (A.Y.O.); gup1.pharmacy@gmail.com (A.G.I.); 3 Faculty of Pharmacy, Kazakh-Russian Medical University, Abylai Khan St. 51/53, Almaty 050004, Kazakhstan; f.kaiupova@medkrmu.kz (F.E.K.); zhumalinaa1962@mail.ru (K.Z.Z.)

**Keywords:** *Cirsium arvense*, phytochemistry, antioxidant activity, antimicrobial activity, ethnomedicinal uses

## Abstract

*Cirsium arvense* (L.) Scop is a perennial plant of the family Asteraceae that is mainly distributed in the temperate regions of the Northern Hemisphere. Despite being widely recognized as an invasive weed in agriculture, most of the scientific evidence shows its significant phytochemical and pharmacological importance. In the present review article, a comprehensive summary of the available literature on *C. arvense’s* botanical properties, phytochemical composition, biological activities, standardization potential, and future therapeutic prospects has been carefully provided. This plant has been used traditionally for the treatment of inflammation, infections, bleeding disorders, and liver-related disorders. Phytochemical investigations showed the presence of many bioactive compounds such as flavonoids, phenolic acids, triterpenes, sterols, tannins, glycosides, and volatile compounds. Among the reported biological activities, antioxidants and antimicrobial properties are the most studied activities. In addition, anticancer, antidiabetic, neuroprotective, anti-inflammatory, and antiproliferative activities have also been investigated. The environmental adaptability, rapid growth, and extensive root system of *C. arvense* highlight its potential for development as a sustainable medicinal and industrial crop. However, there are critical research gaps present in phytochemical standardization, toxicity assessment, pharmacokinetics, and clinical validation, warranting further comprehensive studies.

## 1. Introduction

Medicinal plants have been recognized as valuable sources of therapeutic properties for centuries and continue to play a fundamental role in traditional and modern medical systems. Since ancient times, various plants have been used to treat various ailments in folk remedies, which formed the basis of such systems as Ayurveda, Traditional Chinese Medicine, and Greek Medicine [1,2]. These plants are rich in diverse bioactive compounds such as alkaloids, flavonoids, phenolic acids, terpenoids, and glycosides, which are responsible for their extensive pharmacological activities. Scientific evidence has shown that these phytochemicals have antioxidant, anti-inflammatory, antimicrobial, antiviral, antidiabetic, and anticancer properties, making medicinal plants extremely important in the prevention and treatment of diseases [3,4,5]. In recent decades, due to the adverse effects of synthetic drugs, high costs, and problems of drug resistance, there has been a renewed focus on medicinal plants. Natural products are generally considered safer, more biocompatible, and more environmentally friendly. Furthermore, it has been estimated that a large proportion of existing drugs are either derived directly from plants or are based on plant compounds, highlighting their importance in drug discovery and development. Advances in phytochemistry, pharmacology, and molecular biology have contributed significantly to our understanding of plant compounds and their mechanisms of action [6,7,8]. However, considering their diverse pharmacological properties and therapeutic potential, comprehensive reviews are essential to facilitate their safety and effective use in modern medicine.

The genus Cirsium (family Asteraceae) consists primarily of perennial thorny herbs that are widespread in the temperate regions of the Northern Hemisphere, including Eurasia, Asia, North America, and North Africa. The name Cirsium is derived from the Greek word “khirsos”, which means “swollen vein”, reflecting its traditional use in the treatment of bleeding disorders and vascular diseases [9,10]. There are approximately 450–480 species of this genus worldwide, many of which are used in traditional medicine, particularly traditional Chinese medicine, to stop bleeding, reduce inflammation, and promote diuresis [10,11,12]. Recent scientific studies have shown that Cirsium species have liver-protective, antioxidant, antimicrobial, and anticancer properties, which is mainly due to bioactive compounds such as flavonoids, phenolic acids, and terpenoids. Differences in the chemical composition of different species also lead to differences in their biological activities, which indicates the need for more systematic studies of this important medicinal species [11,13].

Among the various species of the genus Cirsium, *Cirsium arvense*, commonly known as Creeping thistle, is one of the most widespread and economically significant species, and it has received special attention due to its ecological adaptability and medicinal value. It is a perennial herb native to Europe and other parts of the Northern Hemisphere, and it has since been introduced into many regions as a very aggressive and persistent weed [13,14]. It grows in a variety of environments, such as dry sandy soils, humid areas, grasslands, and disturbed or uncultivated areas, and thrives best in temperate climates. A notable characteristic of this species is its robust vegetative system, which includes an extensive network of horizontal and vertical roots, which gives it the ability to spread rapidly through both seed and vegetative growth and increases its environmental robustness [12,15]. *C. arvense* can reach heights of approximately 150–180 cm, and its deep and branching roots allow continuous growth by storing nutrients. Although it is considered an invasive herb, its medicinal value has been recognized for centuries, and it has been traditionally used to treat bleeding, stomach ailments, skin diseases, infections, and high blood pressure [16,17]. Phytochemical studies have shown that it contains flavonoids, phenolic acids, triterpenoids, sterols, and other biologically active compounds, which are responsible for its antioxidant, anti-inflammatory, antimicrobial, antidiabetic, and anticancer properties [18,19]. Along with this, its abundance of bioactive compounds and traditional medicinal properties have attracted increasing interest in scientific research, further leading to *C. arvense* being viewed as a potential source of nutraceuticals, pharmaceuticals, and functional foods; however, its invasive nature presents some challenges.

The main objective of this review article is to provide a comprehensive overview of *C. arvense*. Particular attention has been given to its traditional uses, phytochemical composition, and pharmacological activities. Furthermore, it seeks to highlight recent research findings, particularly regarding emerging applications in pharmaceuticals and nutraceuticals. Additionally, this review addresses current research gaps, such as the lack of clinical validation, standardization problems, and a limited understanding of the underlying mechanism involved, in order to give guidance for future research. Ultimately, this review provides a better understanding and rationale for the use of *C. arvense* as a promising natural source of novel therapeutic agents.

## 2. Methodology

This study was conducted as a narrative review aimed at providing a comprehensive overview of the current knowledge on *Cirsium arvense* (L.) Scop., including its botanical characteristics, traditional uses, phytochemical composition, biological activities, and future therapeutic potential. A comprehensive literature search was performed using major scientific databases, including Web of Science, Scopus, PubMed, ScienceDirect, and Google Scholar.

The literature search was performed between March and May 2026, with the final search completed in May 2026. Search terms were used individually and in combination using Boolean operators (AND/OR), including “*Cirsium arvense*”, “creeping thistle”, “Canada thistle”, “medicinal plants”, “traditional uses”, “phytochemistry”, “secondary metabolites”, “biological activity”, “pharmacological activity”, “antioxidant”, “antimicrobial”, “anti-inflammatory”, “hepatoprotective”, “anticancer”, and “therapeutic potential”. Examples of search strings include: (“*Cirsium arvense*” OR “Canada thistle”) AND (“phytochemistry” OR “secondary metabolites”), and (“*Cirsium arvense*”) AND (“biological activity” OR “pharmacological activity”).

Peer-reviewed research articles, review papers, books, book chapters, and authoritative reports published in English up to May 2026 were considered. Studies were selected based on their relevance to the objectives of this review, with particular emphasis on botanical characteristics, ethnomedicinal applications, phytochemical constituents, biological activities, safety aspects, and potential pharmaceutical applications of *C. arvense*. Publications lacking sufficient scientific information, duplicate records, conference abstracts, and non-English sources were excluded.

The retrieved literature was critically evaluated and organized into thematic sections to provide an updated synthesis of the available evidence, identify current knowledge gaps, and highlight future research directions for *C. arvense*.

## 3. Botanical Description and Taxonomy

*C. arvense* (L.) Scop., commonly known as creeping thistle or Canadian thistle, belongs to the family Asteraceae (subfamily Carduoideae), and is the most economically important weed species in temperate regions globally. According to the Plants of the World Online (POWO) database, the genus “Cirsium” currently contains 498 recognized species and is widely distributed in temperate regions of the Northern Hemisphere, including Europe, Asia, North Africa, and North America [9,20]. However, despite being classified as a harmful weed in many agricultural systems, recent studies based on ethnopharmacological and phytochemical data have shown notable therapeutic potential, presenting a review of its botanical and economic status [13,21,22].

**Figure 1:**
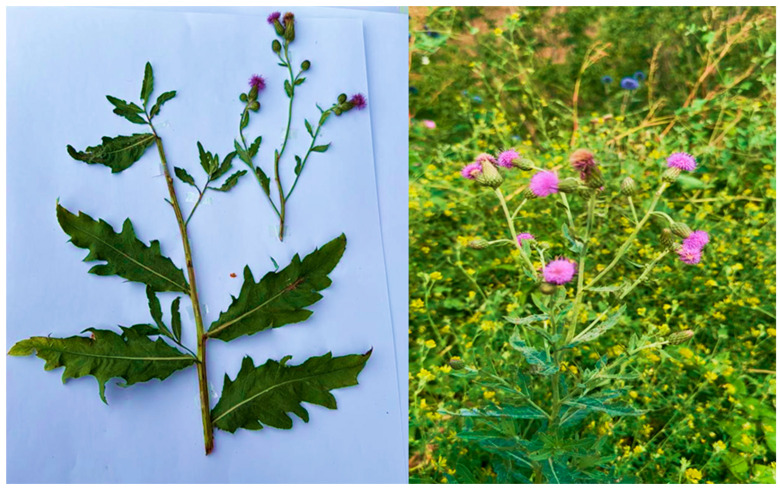
Morphological characteristics of *C. arvense* showing the vegetative and flowering parts of the plant. The left panel presents the collected specimen, displaying the stem and deeply lobed spiny leaves, while the right panel illustrates the natural habitat and characteristic purple capitula (flower heads) of *C. arvense* in the field. Voucher specimen No. 9462 was collected from the Karasai district, Almaty region, Kazakhstan (43.163348° N, 76.753390° E; 860 m altitude) on 23 June 2025 and deposited in the herbarium collection of the Institute of Plant Biology and Biotechnology, Kazakhstan.

*C. arvense* is described as a perennial herb, which shows unique adaptability in a variety of ecosystems and agricultural settings. It is a perennial, dioecious plant that can grow typically up to 160 cm tall, with an erect, unwinged and branched stem. The leaves of *C. arvense* are petiolate and ovate to oblong (3–30 × 1–6 cm), with smoother or slightly recurved margins and small spines (1–7 mm). Its inflorescence is a capitulum mainly containing reddish-purple or sometimes white flowers, while the fruit is a yellowish achene with a white hairy pappus [13,15]. Its ecological success is based on its robust root system, which has a high capacity for storing carbohydrates, and on efficient vegetative propagation methods that give it the ability to spread and regenerate quickly. Unlike other invasive species, the spread of *C. arvense* is largely influenced by human activities, especially agricultural intensification, irrigation systems, and climate change [15,23]. Figure 1 shows the morphological characteristics and natural habitat of *C. arvense*, including its deeply lobed leaves and characteristic purple flower heads.

## 4. Historical and Ethnobotanical Context

The genus Cirsium has been medicinally important for centuries, and it has a prominent place in various traditional medical systems, particularly Traditional Chinese Medicine (TCM). The earliest record of its use is found in the famous book Famous Doctor Records from the Han Dynasty, where the plants are described as having properties that help “stabilize pregnancy, prevent hematemesis, reduce bleeding and relieve physical weakness” [11]. Over time, various species of this genus, including *C. arvense*, have been widely used in traditional medicine in various regions of the world, such as China, Japan, Europe, and the Middle East. These plants have been considered particularly useful in the treatment of bleeding disorders, inflammatory diseases, skin problems, and infectious diseases, which demonstrates their multifaceted therapeutic value [13,18,24,25].

Although traditional uses vary among regions and cultures, several repeated applications related to the treatment of inflammatory conditions, bleeding disorders, and general health support have been reported for *C. arvense* and other members of the genus Cirsium. In Turkey and parts of the Middle East, leaves of *C. arvense* and other edible wild thistles have been traditionally used in dishes such as sarma (cooked leaves wrapped with rice and meat filling), a well-known traditional dish of these regions; this demonstrates the plant’s status as both a food and a medicine [26]. In Northern Europe, especially Estonia, ancient evidence shows that *C. arvense* was used as an ingredient in soups, porridges, and breads during times of famine, suggesting its nutritional value and medicinal properties. The convergence of these traditional uses across diverse geographical and cultural regions provides strong ethnobotanical evidence for plant bioactive properties [19,27].

## 5. Weed Status, Ecological Role, and Resource Potential of *C. arvense*

The classification of plants as “weed” or “cultivated plants” is a continuum that is mainly based on decisions rooted in human use and context, not on a plant’s natural botanical characteristics [28,29]. *C. arvense* is a clear example of this concept. However, it is considered a highly competitive and economically damaging perennial weed in temperate agricultural systems, causing significant yield losses in both organic and conventional crops; it has exceptional adaptability and the ability to grow in a variety of environments without external inputs, such as irrigation or fertilizer, which also gives it the potential to be reimagined as a versatile biological resource [30,31]. Its rapid spread, enabled by its extensive root system and efficient vegetative propagation, makes it difficult to control and requires integrated strategies based on a combination of chemical and agronomic methods [15,32]. The use of herbicides raises environmental and sustainability concerns, leading to a growing interest in non-chemical and integrated control methods. Even with these challenges, *C. arvense* also offers important ecological benefits and serves as a host for numerous insects, particularly bees, attracting pollinators with its abundant nectar and fragrant flowers. These ecological services, along with its proven medicinal and nutritional value, challenge traditional perception and suggest that it should be viewed not only as a harmful weed but also as a valuable biological resource [17,23,33]. Table 1 shows the scientific classification, distribution, habitat, traditional use, main phytochemicals, and biological activities of *C. arvense*.

Although *C. arvense* has medicinal and economic potential, it still exists as an invasive weed in many agricultural systems. Its extensive root system, vegetative reproduction, and efficient seed dispersal play important roles in its rapid spread and persistence. Therefore, any future cultivation or commercial use of *C. arvense* must incorporate appropriate management, containment, and biosecurity measures to minimize environmental and agricultural risks and ensure sustainable use.

## 6. Phytochemical Composition

### 6.1. Flavonoid Profile and Bioactive Glycosides

**Figure 2:**
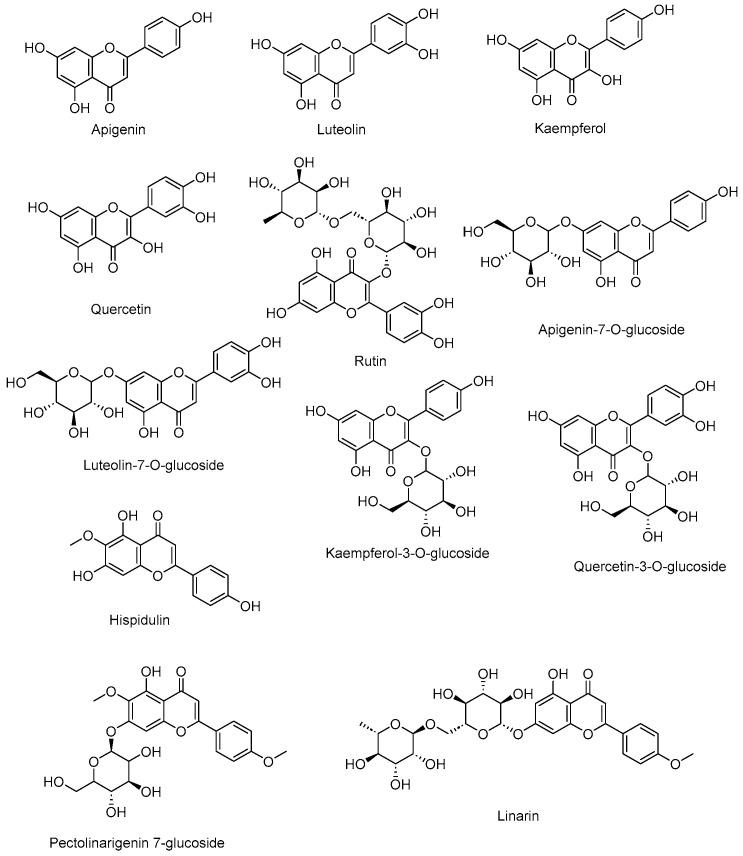
Structures of flavonoids and flavonoid glucoside found in *C. arvense*.

The phytochemical composition of *C. arvense* reveals unique biochemical complexity, with flavonoids appearing as a prominent and dominant group of secondary metabolites and playing a fundamental role in most of its therapeutic activities. The plant is particularly rich in flavones and flavonols, which further enhance its biological efficacy. The identified flavonoids in *C. arvense* include apigenin, luteolin, quercetin, kaempferol, rutin, hispidulin, linarin, pectolinarigenin-7-glucoside, apigenin-7-O-glucoside, luteolin-7-O-glucoside, kaempferol-3-O-glucoside, and quercetin-3-O-glucoside, which display various biological properties and play a significant role in its pharmacological effects. These compounds were identified using High-Performance Liquid Chromatography coupled with Diode Array Detection (HPLC-DAD), isolated through repeated chromatographic separations, including vacuum liquid chromatography and other conventional purification techniques [19,34,35,36,37]. The structures of these compounds are shown in Figure 2.

### 6.2. Triterpenes, Sterols, and Lipophilic Components

**Figure 3:**
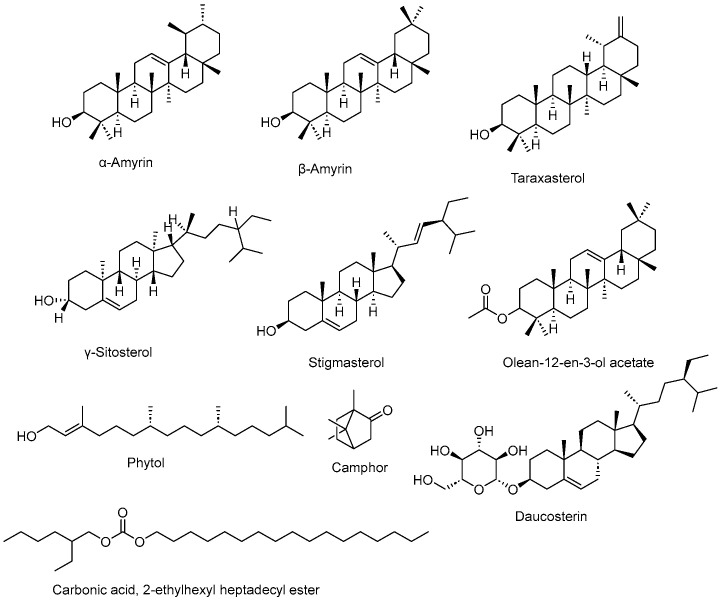
Structures of triterpenes, sterols, and lipophilic components in *C. arvense*.

*C. arvense* contains a diverse range of triterpenes, sterols, and other lipophilic constituents that contribute significantly to its biological and pharmacological activities. Phytochemical studies have identified triterpenoids such as α- and β-amyrin and taraxasterol, particularly in the methanolic extracts of the aerial parts and flowers. Sterols including γ-sitosterol and stigmasterol have also been reported, along with compounds such as olean-12-en-3-ol acetate. In addition, several lipophilic molecules such as fatty acids, their methyl esters, phytol, camphor, and other non-polar constituents have been detected through GC-MS analysis [12,13,38]. A steroid glucoside known as daucosterin and long-chain esters including carbonic acid 2-ethylhexyl heptadecyl ester have been detected in *C. arvense* by using different chromatographic techniques [36,39]. The structures of these reported compounds are shown in Figure 3.

### 6.3. Phenolic Acids and Hydroxybenzoic Acid Derivatives

**Figure 4:**
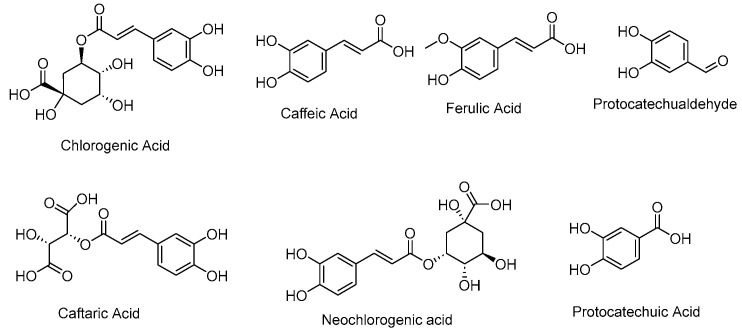
Structures of phenolic acids and hydroxybenzoic acid derivatives.

Phenolic acids constitute a critical component of *C. arvense* phytochemistry, with chlorogenic acid, caffeic acid, and ferulic acid indicating the predominant structures. Comparative studies within the genus Cirsium indicate that phenolic acid composition may vary among species and according to environmental and seasonal factors. However, detailed investigations specifically addressing these variations in *C. arvense* remain limited [19,40]. In addition to this, caftaric acid and neochlorogenic acid are also prominently reported. Finally, hydroxybenzoic acid derivatives, including protocatechuic acid and protocatechualdehyde, have been detected in different parts of *C. arvense* [13,34]. The structures of these compounds are shown in Figure 4.

### 6.4. Volatile and Related Phytochemical Constituents

**Figure 5:**
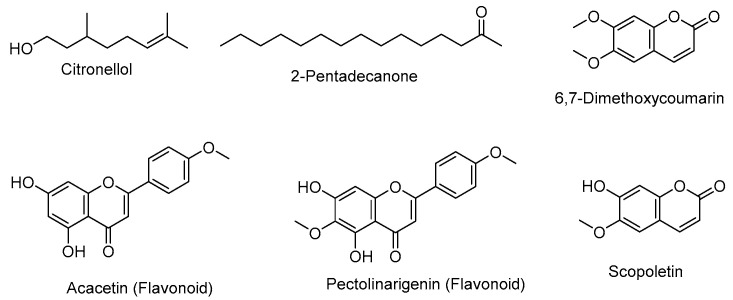
Structures of representative volatile and related phytochemical constituents reported in *C. arvense*.

*C. arvense* contains a range of volatile and semi-volatile constituents that contribute to its characteristic biological activity. These include compounds such as citronellol and 2-pentadecanone, along with aromatic derivatives like benzopyran and coumarin compounds (e.g., 6,7-dimethoxycoumarin). These compounds were detected through GC-MS analysis [38]. In addition, flavonoid-related derivatives such as acacetin and pectolinarigenin glycosides, as well as scopoletin, have been reported as part of the plant’s volatile-associated phytochemical profile. These compounds are known to play important roles in plant defense, antimicrobial activity, and ecological interactions, including pollinator attraction. Although reports on polyacetylenes in *C. arvense* are limited, related compounds within the genus suggest their possible contribution to the plant’s bioactivity [13,41]. The structures of the compounds are shown in Figure 5.

**Figure 6:**
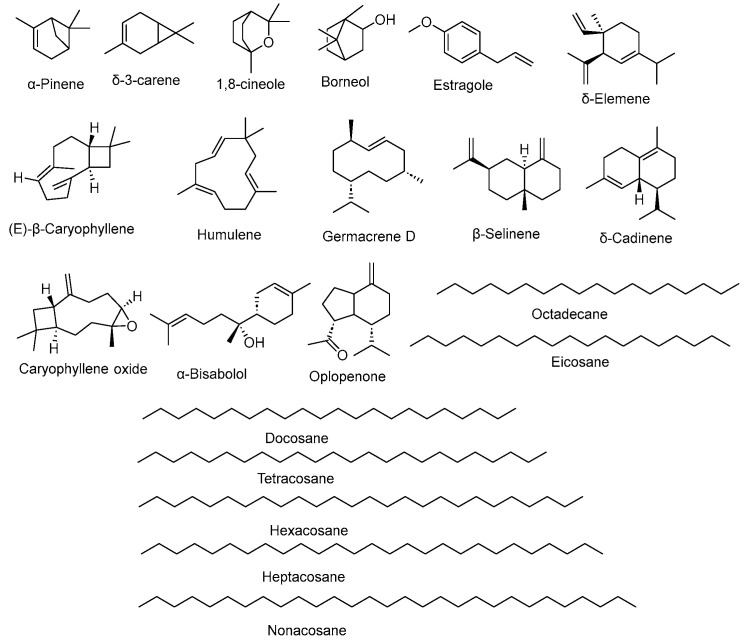
Structures of essential oil reported in *C. arvense*.

Furthermore, Ali Dehjurian et al. showed that *C. arvense* contains a diverse range of essential oil and volatile constituents, which were determined through GC-MS analysis. The oil extracted amount was 0.25, 0.41, and 0.25 (*w*/*w* %) for flower, stem, and leaf, respectively. These essential oil and volatile constituents include monoterpenes such as α-pinene, δ-3-carene, 1,8-cineole, camphor, borneol, and estragole, as well as sesquiterpenes like δ-elemene, E-β-caryophyllene, α-humulene, germacrene D, β-selinene, δ-cadinene, and caryophyllene oxide. Additionally, oxygenated compounds such as α-bisabolol and oplopenone have been found as major compounds in some extracts. It also contains various aliphatic hydrocarbons and long-chain compounds, including octadecane, eicosane, docosane, tetracosane, hexacosane, heptacosane, and nonacosane, along with bioactive diterpenoids such as phytol and other lipophilic constituents. The major constituents of the flower oil were α-bisabolol (17.4%), hexacosane (12.6%), and δ-cadinene (9.7%), whereas leaf oil was dominated by α-bisabolol (34.8%), δ-cadinene (20.7%), and β-selinene (15.6%). In stem oil, α-bisabolol (45.8%) and δ-cadinene (23.2%) were identified as the predominant compounds [42]. The structures of these compounds are shown in Figure 6. Table 2 shows the reported phytochemicals in *C. arvense*.

### 6.5. Critical Evaluation of the Phytochemical Profile and Future Perspectives

Phytochemical investigations conducted so far have shown that *C. arvense* possesses a chemically diverse metabolite profile, mainly containing flavonoids, phenolic acids, triterpenes, sterols and volatile compounds. Of these classes, flavonoids and phenolic acids are the most consistently reported compounds across studies and are likely responsible for the observed antioxidant and antimicrobial activities. However, substantial variability in phytochemical composition has been observed depending on different plant parts, extraction methods, geographical origin, and environmental conditions, making direct comparisons between different studies difficult.

An important observation from the available literature is that most phytochemical studies have focused only on the identification of major compounds, while comprehensive metabolomic characterization is still limited. As a result, the specific metabolites responsible for individual pharmacological effects are not yet fully understood. Therefore, future investigations should prioritize bioactivity-guided isolation, quantitative phytochemical profiling, and structure–activity relationship (SAR) investigations to identify important therapeutic compounds.

Recent advances in metabolomics, high-resolution mass spectrometry, molecular networking, and integrated omics technologies provide powerful tools for comprehensive identification of metabolites of *C. arvense*. Application of these methods may be helpful in the discovery of new bioactive compounds, improvement of phytochemical standardization, and achieving a deeper understanding of biosynthetic pathways and chemical variation within the species. Such information will be essential for the formulation of quality control standards, reproducible herbal preparations, and future pharmaceutical applications of *C. arvense*.

## 7. Biological and Pharmacological Activities of *C. arvense*

### 7.1. Antioxidant Potential of C. arvense

Antioxidants are biologically active compounds that protect biological systems from oxidative stress by neutralizing free radicals and reactive oxygen species (ROS). Oxidative stress is closely linked to the development of various chronic diseases, such as cancer, cardiovascular disease, neurodegenerative diseases, and inflammatory disorders. Natural antioxidants derived from plants such as phenols, flavonoids, and tannins play an important role in eliminating free radicals, chelating metal ions, and preventing lipid oxidation. Due to their efficacy and relatively few side effects, plant antioxidants are gaining increasing importance in pharmaceuticals, nutraceuticals, and functional foods [44,45,46].

The antioxidant potential of *C. arvense* has been extensively studied, which is attributed to its rich phytochemical composition. Various studies have shown that extracts of different parts of this plant exhibit significant antioxidant activity through different mechanisms. For example, root extracts showed significant DPPH radical scavenging activity, which was higher than that of standard antioxidants such as α-tocopherol and BHT at high concentrations, while FRAP and superoxide anion scavenging activities were also significant [36]. Comparative studies on different Cirsium species, including *C. arvense*, also showed that antioxidant capacity was closely related to total phenolic content, where total antioxidant status ranged from 2.31 to 2.78 mM/L as measured by ABTS assay [47]. Furthermore, methanolic flower extracts of *C. arvense* showed remarkable antioxidant activity (TAS = 2.2 mM/L) and high total phenolic content (192 mg/g), while different solvent fractions also showed significant antioxidant capacity, with the n-butanol fraction showing the highest TAS value (2.69 mM/L) [48]. These findings further support the contribution of phenolic compounds to the antioxidant properties of *C. arvense*.

Furthermore, various in vitro assays such as DPPH, nitric oxide, hydrogen peroxide, and superoxide scavenging tests have shown that ethanolic extracts of *C. arvense* possess dose-dependent antioxidant activity, which is mainly due to the presence of phenolic compounds such as quercetin, rutin, kaempferol, and p-coumaric acid [43]. Quercetin was found to be particularly abundant among these compounds, which plays a major role in its antioxidant activity. Furthermore, the highest antioxidant activity was observed in methanolic and ethanolic extracts of leaves and flowers, which is associated with high levels of chlorogenic acid and luteolin derivatives [19]. Furthermore, *C. arvense* has shown significant in vivo antioxidant effects not only in vitro but also in hypercholesterolemic animal models. Methanol extract of this plant significantly increased antioxidant defense indicators, such as total antioxidant capacity (TAC), superoxide dismutase (SOD), catalase, paraoxonase and arylesterase activities, while reducing oxidative stress indicators such as total oxidant status (TOS) and malondialdehyde (MDA). Moreover, this extract also improved the lipid profile, resulting in a decrease in total cholesterol, LDL, and triglyceride levels, while there was an increase in HDL levels, indicating its potential utility in the management of metabolic disorders associated with oxidative stress [49]. Along with this, *C. arvense* extracts showed significant antioxidant effects in quail studies, where MDA levels were reduced in serum, liver, and egg yolk, while the activity of antioxidant enzymes such as SOD, catalase, and Glutathione peroxidase (GSH-Px) was increased. These results indicate that *C. arvense* as a dietary supplement has the potential to reduce oxidative stress and improve the antioxidant defense system [50].

Although the antioxidant results are promising, several limitations and deficiencies still exist in the current literature. Most studies have used crude extracts using different extraction solvents and experimental conditions, making direct comparisons between studies difficult [36,43,48]. Likewise, the role of individual phytochemicals in the overall antioxidant activity is not fully recognized, and synergistic interactions between flavonoids, phenolic acids, and other metabolites may play an important role [19]. Although initial in vivo studies have shown beneficial effects on biomarkers of oxidative stress [49,50], the underlying molecular mechanisms, bioavailability, pharmacokinetic behavior, and long-term efficacy of these compounds remain largely unexplored [51]. Table 3 shows the antioxidant activities of *C. arvense*.

Future investigations should focus on standardized extracts, bioactivity-guided fractionation, and mechanism-based studies to better understand the therapeutic significance of the antioxidant properties of *C. arvense*.

### 7.2. Antimicrobial Potential of C. arvense

Antimicrobial agents from medicinal plants have received special scientific attention in recent years, mainly due to the increasing number of bacteria with antibiotic resistance and the limited effectiveness of conventional antimicrobial drugs. Biologically active compounds obtained from plants, such as flavonoids, phenolic acids, terpenoids, alkaloids, and tannins, show broad antimicrobial activities against various bacteria, fungi, and other pathogenic microorganisms. These compounds exert their activity by damaging the bacterial cell membrane, inhibiting protein and nucleic acid synthesis, and interfering with important metabolic pathways [52,53].

*C. arvense* has demonstrated significant antimicrobial activity against various bacterial and fungal pathogens. Preliminary research on aquatic extracts of Cirsium species showed significant antimicrobial activity, with low-molecular-weight phenolic compounds being more effective than tannins [47]. A comprehensive review of the antimicrobial activity of Cirsium species revealed that flavonoids, triterpenoids and phenolic acids are the major bioactive components responsible for the antimicrobial activity. These genus-level observations may partially explain the antimicrobial activity reported for *C. arvense* [21]. These compounds were found to be effective against major pathogens such as *Acinetobacter baumannii, Pseudomonas aeruginosa* and *Enterococcus faecium*. Possible mechanisms of its antibacterial activity include damage to the bacterial cell membrane and internal structure, inhibition of protein and nucleic acid synthesis, and suppression of energy-generating metabolic pathways [21].

Several experimental studies have evaluated the antibacterial and antifungal activities of extracts obtained from different parts of *C. arvense*. Methanolic extracts of leaves, stems, roots, and flowers showed varying degrees of antifungal activity against *Macrophomina phaseolina*, with leaf extracts being the most effective and reducing fungal biomass by up to 74%. GC-MS analysis of active leaf extracts reported the presence of compounds such as 10-octadecanoic acid methyl ester, benzopyran derivatives, hexadecanoic acid methyl ester, and 9,12-octadecadienoic acid methyl ester, which were attributed to possible antifungal activity [38]. Similarly, different fractions of methanolic extracts have also shown significant antibacterial activity against Gram-positive and Gram-negative bacteria, such as *Staphylococcus aureus, Escherichia coli, Pseudomonas aeruginosa, Klebsiella pneumoniae,* and *Micrococcus luteus*. Among these fractions, the chloroform fraction was found to be the most effective, especially against Staphylococcus aureus, while n-hexane and chloroform fractions also showed strong antifungal activity against *Aspergillus niger* (Table 4) [54].

Recent studies have also combined *C. arvense* extracts with conventional antibiotics to evaluate their combined efficacy against resistant bacteria. The combination *of C. arvense* extracts and cefixime showed full or partial synergistic effects against methicillin-resistant *Staphylococcus aureus* (MRSA), *Escherichia coli* and *Acinetobacter baumannii*. Time-kill kinetic analyses showed a significant decrease in bacterial growth and protein content, indicating that plant extracts may enhance the efficacy of antibiotics [55]. Furthermore, extracts of the aerial parts of *C. arvense* showed dose-dependent antibacterial activity against various pathogenic bacteria [56]. Overall, these results indicate that *C. arvense* has significant antimicrobial potential and may be used as a natural antimicrobial agent or adjunctive therapy in the treatment of resistant bacterial infections in the future. Table 4 shows the antimicrobial activities of *C. arvense*.

The antimicrobial activity of *C. arvense* appears to be the result of the combined action of multiple phytochemical classes rather than a single active compound. Flavonoids and phenolic acids can disrupt microbial membrane integrity, alter membrane permeability, inhibit essential enzymes, and interfere with nucleic acid and protein synthesis. Similarly, terpenoids and volatile compounds may contribute to antimicrobial efficacy through membrane destabilization and inhibition of cellular respiration [21,53]. The synergistic effects observed between *C. arvense* extracts and traditional antibiotics further suggest that these phytochemicals may enhance antimicrobial susceptibility of microbes via multiple complementary mechanisms [55].

Although the antimicrobial results are promising, the available evidence is still fragmented and highly dependent on the extraction method, plant origin, and experimental conditions [21,34,54]. Considerable variation in antimicrobial potency has been found between different plant parts and solvent extracts, making direct comparisons between studies difficult [38,54]. Furthermore, only a limited number of studies have identified specific compounds responsible for antimicrobial activity, and information on their pharmacokinetics, safety, and therapeutic efficacy is lacking [21]. Future research should focus on bioactivity-guided isolation of active ingredients, standardized antimicrobial testing protocols, mechanism-based investigations, and evaluation of their potential use as adjuncts to conventional antimicrobial therapies.

**Table 4:** Antimicrobial activities of *C. arvense*.

Extract/Fraction	Plant Part	Target Organism	Activity	Key Findings	Ref.
Methanol extract	Leaves	*Macrophomina phaseolina*	Antifungal	10–74% biomass reduction	[38]
Methanol extract	Stem	*M. phaseolina*	Antifungal	6–57% biomass reduction	[38]
Methanol extract	Root	*M. phaseolina*	Antifungal	11–39% biomass reduction	[38]
Chloroform fraction	Whole plant	*Staphylococcus aureus*	Antibacterial	MIC = 0.312 mg/mL	[54]
Various fractions	Whole plant	*E. coli*, *P. aeruginosa*, *K. pneumoniae*	Antibacterial	Variable activity	[54]
n-Hexane fraction	Whole plant	*Aspergillus niger*	Antifungal	Strongest antifungal fraction	[54]
Aerial extract + cefixime	Aerial parts	MRSA, *E. coli*, *A. baumannii*	Synergistic	Enhanced antibiotic efficacy	[55]
Aerial extract	Aerial parts	Various bacteria	Antibacterial	Dose-dependent inhibition	[56]

### 7.3. Other Biological Activities of C. arvense

In addition to its antioxidant and antimicrobial properties, *C. arvense* has been reported to exhibit several other biological activities, including anticancer, antidiabetic, neuroprotective, anti-inflammatory, and antiproliferative activities [10]. However, scientific knowledge regarding these pharmacological activities is still relatively limited and scattered. More comprehensive research is needed to better understand their therapeutic potential, underlying mechanisms, safety aspects, and potential applications in pharmaceutical and critical medical fields.

Recent studies have also highlighted the acaricidal activity of *C. arvense*, particularly against *Rhipicephalus microplus*, an important tick species that causes economic losses in livestock. Biological experiments showed that consumption of *C. arvense* significantly increased larval mortality and reduced tick reproductive capacity. Furthermore, in silico molecular docking analysis studies have demonstrated that compounds such as apigenin-7-O-glucoside and pectolinarigenin-7-glucoside exhibit strong inhibitory interactions with the tick protein Subolesin, making them promising candidates as environmentally friendly alternatives to synthetic acaricides [37].

**Figure 7:**
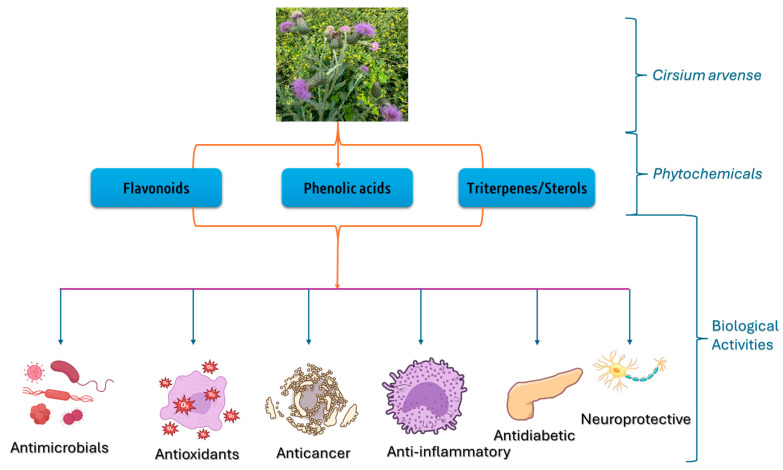
Schematic representation of the phytochemicals and biological activities of *C. arvense*.

Similarly, extracts of *C. arvense* and related species of the family Asteraceae showed significant antiproliferative activity against various cancer cell lines such as HeLa, A431 and MCF7, showing its potential anticancer properties [57]. These effects were more closely related to the inhibition of cell proliferation than to direct cytotoxicity, suggesting the presence of potentially biologically active compounds. Overall, available studies suggest that *C. arvense* contains bioactive compounds with various biological activities. However, compared to its antioxidant and antimicrobial properties, evidence supporting anticancer, antidiabetic, neuroprotective, anti-inflammatory, antiproliferative and acaricidal activities is still limited. Therefore, further pharmacological, toxicological and mechanism-based investigations are needed before definitive conclusions can be drawn about their therapeutic potential. Figure 7 illustrates the biological activities reported for *C. arvense*, particularly.

It is worth noting that a large part of the currently available pharmacological evidence for *C. arvense* has been obtained from in vitro assays, evaluation of crude extracts, in silico analyses, and a limited number of animal studies. Although these results provide valuable preliminary insights into the biological potential of the plant, they do not necessarily translate into clinical efficacy. Therefore, reported pharmacological effects should be viewed with caution until they are supported by standardized preclinical studies, detailed mechanistic investigations, and well-designed clinical trials.

## 8. Future Perspectives: Research Gaps, Development Opportunities, and Strategic Outlook

### 8.1. Critical Research Gaps and Priorities

Despite the growing pharmacological evidence for *C. arvense*, many important research gaps remain, which hinder its effective medical and clinical application. Most of the current studies are based on the crude extracts, while the standardized quantitative and qualitative identification of biologically active compounds is still poor. Therefore, the use of advanced analytical techniques such as HPLC-MS and UPLC-HRMS is essential for the systematic identification and standardization of phytochemical components obtained from different geographic areas, environmental conditions, and harvest times [58,59].

Furthermore, important aspects of pharmacokinetics and pharmacodynamics such as absorption, distribution, metabolism, and elimination are not yet fully understood. A better understanding of these factors is needed for therapeutic efficacy, correct dosing, predicting potential drug interactions, and safety assessment. In addition, although various biological activities of the plants have been reported. Further in-depth studies are needed to elucidate molecular mechanisms and signaling pathways in detail [51].

An additional limitation in the recent literature is the lack of comprehensive toxicological and safety assessments. Although available studies generally report beneficial biological activities and no apparent acute adverse effects, the overall safety profile of *C. arvense* is still poorly defined. Information on its toxicity, such as acute and chronic toxicity, hepatotoxicity, nephrotoxicity, mutagenicity, genotoxicity, reproductive toxicity, herb-drug interactions, and long-term safety, is mainly unavailable. Furthermore, no clear safety margins, maximum tolerated doses, or no-observed-adverse-effect levels (NOAELs) have been established for *C. arvense* extracts or their major bioactive components. This information is essential for dose optimization, risk assessment, and safe translation of experimental results into clinical application. Therefore, systematic toxicological studies in accordance with internationally accepted safety assessment guidelines are essential before clinical development, widespread therapeutic use, and regulatory approval of products derived from *C. arvense* [51,60,61].

Another major limitation of the available literature is that a large part of the phytochemical and pharmacological knowledge has been derived from studies on other Cirsium species rather than *C. arvense*. Although comparative information from closely related species can provide useful insights, species-specific differences in phytochemical composition and biological activity are well recognized. Therefore, direct extrapolation of results from other members of the genus should be viewed with caution, and future investigations should prioritize building evidence specifically for *C. arvense* [10,11,19].

A critical review of the available literature displays a significant imbalance in current research efforts. While antioxidant and antimicrobial activities have been extensively investigated and supported by numerous independent studies [19,21,36,43,47,48,49,50,55,56,57], evidence for other pharmacological properties such as anticancer, neuroprotective, antidiabetic, hepatoprotective, and anti-inflammatory activities is relatively limited [12,13,37,56,57]. In many cases, these biological effects have only been evaluated in preliminary experimental models, making it difficult to establish their definitive therapeutic significance. This discrepancy highlights the need to prioritize mechanism-based and disease-specific investigations to prove the broader pharmacological potential of *C. arvense*.

An important observation is that many biological activities have been attributed to crude extracts rather than to well-identified individual compounds [13,19,36,43]. Although flavonoids, phenolic acids, and triterpenes are generally considered to be the primary bioactive components [10,19,40,41], their relative contributions and potential synergistic interactions are still poorly understood.

Future investigations that combine approaches from phytochemistry, metabolomics, molecular pharmacology, and systems biology may help to explain compound-activity relationships and facilitate evidence-based therapeutic development [44,58,62].

### 8.2. Standardization and Quality Control

As many species of the genus Cirsium have been used in traditional medicine, a comprehensive standardization and quality control system for *C. arvense* has not yet been fully developed. Currently, only *C. japonicum* and *C. setosum* appear in the 2020 edition of the Chinese Pharmacopoeia with an established quality control system [11]. The lack of standardized guidelines for plant identification, geographical origin, harvesting conditions, processing methods, and storage practices can lead to significant variations in phytochemical composition, therapeutic efficacy, and product safety. Such inconsistencies are a major obstacle to the widespread acceptance of this plant in medicinal and clinical settings.

Therefore, it is essential to implement a strong quality control system to ensure the safety, efficacy, and reproducibility of *C. arvense*-based products. Standardization procedures should include accurate plant identification, selection of appropriate medicinal parts, improvement of extraction methods, and the use of advanced chromatographic and spectroscopic techniques for the quantitative testing of marker compounds [62]. Furthermore, phytochemical fingerprinting can be an effective strategy for assessing the authenticity and identity of plant materials, while also considering natural biodiversity. The development of standard pharmacopeial monographs and regulatory frameworks for *C. arvense* will play a crucial role in promoting its scientific validation and future medical use [60,61].

From a translational perspective, the successful development of products derived from *C. arvense* will depend not only on phytochemical standardization but also on the establishment of a robust safety, efficacy and regulatory framework [60,61]. The growing global interest in plant-based pharmaceuticals, nutraceuticals and functional foods presents a significant opportunity for the commercialization of *C. arvense* [1,6,61]. However, achieving this goal will require multidisciplinary collaboration between phytochemists, pharmacologists, toxicologists, clinical experts and regulatory authorities to bridge the gap between traditional uses, experimental evidence and clinical application [51,60,61].

## 9. Conclusions

*C. arvense* is an important but relatively under-researched medicinal plant with considerable ethnopharmacological, phytochemical and therapeutic potential. Although it is known as an invasive and economically damaging plant in agricultural systems, the growing evidence shows that *C. arvense* is a rich source of phytochemicals, such as flavonoids, phenolic acids, triterpenes, sterols, and volatile compounds, which play a vital role in its diverse biological activities. In traditional medicinal systems across various geographic regions, this plant has been used to treat bleeding disorders, inflammation, infection, liver diseases, and many other ailments, representing its historical medicinal value.

Among the reported biological activities, antioxidant and antimicrobial activities are the most extensively studied and scientifically validated. Both of these activities are attributed to the phytochemical richness of this plant. Additionally, preliminary findings suggest hepatoprotective, antidiabetic, anticancer, acaricidal, and neuroprotective activities, although more detailed scientific research and validation of these properties are necessary.

Despite the growing interest in *C. arvense*, several important challenges remain. Comprehensive phytochemical standardization, quality control systems, pharmacokinetic profiling, toxicity assessment, molecular mechanism studies, and systematic clinical investigation are still lacking. Therefore, future research should focus on multidisciplinary approaches consisting of phytochemistry, molecular biology, pharmacology, toxicology, biotechnology, and clinical science to fully elucidate the medicinal value of this plant.

Interestingly, the same biological characteristics that make *C. arvense* a robust and tenacious weed—its exceptional adaptability, extensive root system, efficient vegetative propagation, and ability to thrive in scare agriculture resources—could prove advantageous in its future domestication as a sustainable medicinal and industrial crop. Additionally, its ecological role in supporting pollinators and biodiversity increases its importance in sustainable agricultural systems.

## Figures and Tables

**Table 1:** Botanical characteristics, distribution, traditional uses, phytochemical constituents, and major biological activities of *C. arvense*.

Parameter	Description
Scientific Name	*Cirsium arvense* (L.) Scop.
Family	Asteraceae
Common Names	Creeping thistle, Canada thistle
Synonyms	*Carduus arvensis* L., *Breea arvensis* (L.) Less.
Plant Type	Perennial herbaceous plant
Growth Habit	Erect, spiny, rhizomatous, highly invasive weed
Height	Approximately 30–160 cm
Distribution	Widely distributed in temperate regions of Europe, Asia, North Africa, and North America
Habitat	Agricultural fields, grasslands, roadsides, disturbed lands, forest edges, and moist to dry habitats
Traditional Uses	Used traditionally for inflammation, bleeding disorders, infections, liver ailments, and skin diseases
Major Phytochemicals	Flavonoids, phenolic acids, triterpenes, sterols, tannins, glycosides, volatile compounds
Major Biological Activities	Antioxidant, antimicrobial

**Table 2:** Reported phytochemical constituents of *C. arvense*.

Phytochemical Class	Reported Compounds	Plant Part/Extract	Reference
Flavonoids	Apigenin, luteolin, quercetin, kaempferol, rutin, hispidulin, linarin, apigenin-7-O-glucoside, luteolin-7-O-glucoside, kaempferol-3-O-glucoside, quercetin-3-O-glucoside, pectolinarigenin-7-glucoside	Aerial parts, leaves, flowers	[18,19,34,35,36,37,40,41]
Phenolic acids	Chlorogenic acid, caffeic acid, ferulic acid, caftaric acid, neochlorogenic acid, protocatechuic acid, protocatechualdehyde, p-coumaric acid	Whole plant, aerial parts	[13,19,40,43]
Triterpenes & Sterols	α-Amyrin, β-Amyrin, taraxasterol, γ-sitosterol, stigmasterol, olean-12-en-3-ol acetate, daucosterin	Flowers, aerial parts	[18,36,38]
Volatile compounds	Citronellol, 2-pentadecanone, camphor, phytol, scopoletin, acacetin, pectolinarigenin	Flower, leaf and stem extracts	[38,42]
Essential oil constituents	α-Pinene, δ-3-carene, 1,8-cineole, borneol, estragole, δ-elemene, E-β-caryophyllene, α-humulene, germacrene D, β-selinene, δ-cadinene, caryophyllene oxide, α-bisabolol, oplopenone	Flower, leaf and stem extracts	[42]
Fatty acids and derivatives	9,12,15-octadecatrienoic acid, hexadecanoic acid methyl ester, 10-octadecanoic acid methyl ester, 9,12-octadecadienoic acid methyl ester	Flowers	[38,39]
Other constituents	Choline, alkaloids, glycosides, saponins, cyanogenic glycosides, tannins, carotenoids, tocopherols	Various plant parts	[12,13,19]

**Table 3:** Antioxidant activities of *C. arvense*.

Plant Part/Extract	Assay	Activity/Result	Positive Control	Reference
Root extract	DPPH	Higher activity than α-tocopherol and BHT at higher concentrations	α-Tocopherol, BHT	[36]
Root extract	FRAP	Strong reducing activity	BHT	[36]
Root extract	Superoxide scavenging	Higher than BHT	BHT	[36]
Methanolic flower extract	TAS	TAS = 2.2 mM/L; TPC = 192 mg/g	-	[48]
n-Butanol fraction (flower extract)	TAS	Highest antioxidant activity (TAS = 2.69 mM/L)	-	[48]
Ethanol extract	DPPH, NO, H_2_O_2_, Superoxide	Dose-dependent activity	Standard antioxidants	[43]
Methanol extract (animal study)	TAC, SOD, CAT, MDA	Improved antioxidant status and lipid profile	Control group	[49]
Dietary extract (quail)	SOD, CAT, GSH-Px, MDA	Increased antioxidant enzymes and reduced MDA	Control group	[50]

## Data Availability

All data about this review article are available in this manuscript; no further data has been generated.
